# Coping Strategies and Relationship with Burnout among Residents in Thailand

**DOI:** 10.1192/j.eurpsy.2023.711

**Published:** 2023-07-19

**Authors:** P. Kusalaruk, J. Saravithi

**Affiliations:** Psychiatry, Faculty of Medicine Ramathibodi Hospital, Bangkok, Thailand

## Abstract

**Introduction:**

Burnout is prevalent in residents and coping is one of the important modifying factors.

**Objectives:**

This study aimed to investigate coping strategies, burnout, and their relationship among residents.

**Methods:**

A cross-sectional descriptive study was conducted among residents from October 2019 to April 2020 in Thailand. The Brief COPE Inventory Thai version and The Maslach Burnout Inventory Thai version were used, and the associations between coping strategies and burnout were examined.

**Results:**

The number of 280 residents replied the questionnaire (response rate 41.5%). The most favored copings were self-distraction and acceptance and the least common were denial and substance use. Most residents had high level of emotional exhaustion (n = 113, 40.4%) and moderate level of reduced personal accomplishment (n = 99, 35.4%). However, low degree of depersonalization was reported predominantly (n = 164, 58.6%). The coping of venting, behavioral disengagement and self-blame independently predicted emotional exhaustion and depersonalization. Behavioral disengagement was the only predictor of burnout in all dimensions, whereas positive reframing is the only strategy that had independent and protective effect against burnout in all dimensions.

**Table 1:** Multivariate analysis of factors associated with emotional exhaustion

**Variables**^**a**^ **associated with emotional exhaustion**	**β (S.E.)**	**95% CI**	**P value**
Venting	.17^**^ (.50)	(.50, 2.46)	< .01
Behavioral disengagement	.29^***^ (.51)	(1.52, 3.55)	< .001
Self-blame	.15^**^ (.47)	(.30, 2.15)	.01
Planning	.15^**^ (.51)	(.32, 2.33)	.01
Positive reframing	-.26^***^ (.50)	(-3.08, -1.12)	< .001

**Table 2:** Multivariate analysis of factors associated with depersonalization

**Variables**^**a**^ **associated with depersonalization**	**β (S.E.)**	**95% CI**	**P value**
Sex	-.12* (.58)	(-2.37, -.07)	.04
Alcohol use	.18^**^ (.75)	(.74, 3.69)	< .01
Venting	.14* (.26)	(.07, 1.11)	.03
Behavioral disengagement	.18^**^ (.25)	(.28, 1.27)	< .01
Self-blame	.21^***^ (.23)	(.38, 1.28)	< .001
Positive reframing	-.18* (.23)	(-1.17, -.27)	< .01
Use instrumental support	-.14* (.24)	(-1.03, -.10)	.02

**Table 3:** Multivariate analysis of factors associated with personal accomplishment

**Variables**^**a**^ **associated with personal accomplishment**	**β (S.E.)**	**95% CI**	**P value**
Behavioral disengagement	-.13* (.38)	(-1.59, -.09)	.03
Positive reframing	.21** (.41)	(.42, 2.03)	< .01

a Only statistically significant variables are displayed

**Conclusions:**

A large number of residents had emotional exhaustion. Behavioral disengagement and positive reframing were the most influential coping strategies related to burnout. This study might inform residency training program of some specific approaches for burnout prevention.

**Disclosure of Interest:**

None Declared

